# YSED: Yemeni speech emotion dataset

**DOI:** 10.1016/j.dib.2025.112233

**Published:** 2025-11-02

**Authors:** Somia Derhem, Eiad AL-Mekhlafi, Nashwan Ahmed AL-Majmar, Moeen AL-Makhlafi

**Affiliations:** a Department of Computer Science and Information Technology, IBB University, IBB, Yemen; b College of Science and Engineering, Aljazeera University, IBB, Yemen; c College of Science and Engineering, National University, IBB, Yemen; d School of Information Engineering, Wuhan College, Wuhan 430212, Wuhan, Hubei, China

**Keywords:** Data science, Speech emotion recognition, Arabic Language, Arabic emotions

## Abstract

Arabic encompasses numerous dialects, including the Yemeni dialect, which is notably underrepresented in speech emotion recognition (SER) research. To support deep learning-based emotion recognition in Yemeni speech, there is a critical need for high-quality audio datasets that capture the emotional nuances specific to Yemeni Arabic. The limited availability of such resources has hindered research progress in this domain, particularly for Arabic and its dialects. In this paper, we introduce the Yemeni Speech Emotion Dataset (YSED), the first of its kind for Yemeni Arabic. YSED comprises audio recordings from 71 Yemeni volunteers (37 males and 34 females) aged between 15 and 45 years, expressing five emotions: anger, sadness, happiness, fear, and neutrality. Each participant recorded five sentences per emotion, with four repetitions for emotional states, and one for neutral, resulting in an initial 6035 sentence recordings. Recording duration ranged from 2 to 7 s using the simulated and induced approach. After evaluation by six judges, a total of 1432 recordings were validated, with an overall duration of 59 min and 10 s. The analysis achieved a Fleiss' Kappa coefficient of 0.9, indicating a high level of inter-annotator agreement. The YSED dataset is expected to be a valuable resource for researchers in emotion analysis and application development, enhancing the processing of Yemeni speech at an emotional level and informing services and social interactions. This work represents a significant step toward enriching data sources for understanding emotions in Yemeni Arabic and other languages and cultures. The dataset will be accessible at the following link: https://zenodo.org/records/15227219.

Specifications Table

**Table:** 

Subject	Computer Sciences
Specific subject area	Arabic Language, Machine Learning, Natural Language Processing, Audio Classification, Automatic Speech Recognition.
Type of data	Speech/audio
Data collection	Private and public universities, public secondary schools, faculty members and employees.
Data source location	City: IBB Country: Yemen
Data accessibility	Repository name: YSED Direct URL to data: https://zenodo.org/records/15227219

## 1 Value of the Data

Emotions are an essential part of human communication and express personal feelings and attitudes.

• The presented dataset is significant because it is the first dataset to recognize Yemeni speech emotions to our knowledge.
• This dataset can contribute to improving communication and understanding social interactions in Yemeni society.
• By applying deep learning techniques to this dataset, an accurate model of emotion recognition can be developed in Yemeni speech, enabling us to achieve many benefits.
• YSED dataset encourages researchers to explore Yemeni speech emotions and experiment with other Yemeni speech SER models.

## 2 Background

Emotion recognition in speech is a critical area in human–computer interaction and affective computing, increasingly driven by machine and deep learning methods [1,2]. Among world languages, Arabic ranks fifth in the number of speakers, yet remains underrepresented in speech emotion recognition (SER) research [3,4]. Existing studies highlight challenges, including limited datasets and dialectal diversity. Iben Nasr et al. [5] provide a comprehensive survey of Arabic SER, identifying issues in emotional modelling and data acquisition. Keleg et al. [6] further emphasize how oversimplified assumptions about dialectal boundaries affect model performance, noting significant overlap between regional dialects. Studies such as Kaloub and Elgabar [7] have explored Arabic SER using small datasets, achieving accuracies up to 83.82 % for basic emotions.

While progress has been made on dialects such as Saudi [8] and Algerian [9], Egyptian [10], Emirati [11], Tunisian [12], and Moroccan [13], research on Yemeni Arabic remains scarce. The Yemeni dialect shares linguistic and phonetic traits with neighbouring dialects, particularly those of Saudi Arabia and Oman, enabling cross-dialectal emotion studies [14,15]. To address this gap, this paper introduces the Yemeni Speech Emotion Dataset (YSED), covering five emotions: happiness, sadness, anger, fear, and neutral.

## 3 Data Description

In this paper, we introduce the Yemeni Speech Emotion dataset (YSED), which comprises 1432 audio recordings from 71 male and female participants aged between 15 and 40 years. The dataset encompasses five emotions: anger, fear, happiness, sadness, and neutral. The dataset is structured into five distinct folders, each corresponding to a specific emotion and containing the associated audio recordings. (YSED) unbalanced dataset in terms of categories with 268 recordings for anger, 249 recordings for happy, 303 recordings for sad, 244 recordings for fear emotions and 305 recordings for neutral emotions, as depicted in Table 1.

**Table 1:** The distribution of recordings by category.

Emotions	Total
Neutral Fear Happy Sad Angry	305 244 249 303 268

**Fig. 1:**
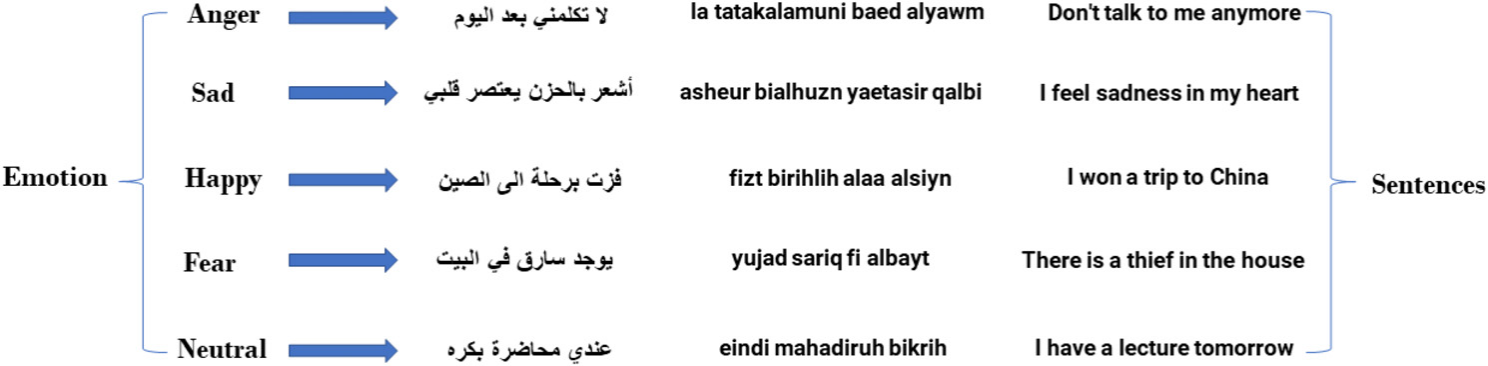
Sample of the sentences that were recorded with the corresponding category.

The recordings were saved in raw format without any cleaning or pre-processing. Five sentences were chosen for each of the five emotions, which means that the total number of sentences for the emotions became (5 × 5 = 25) sentences. Fig. 1 shows a sample of the sentences chosen for each emotion.

By reading a lot of literature related to emotion recognition, we compared some datasets with our dataset. Table 2 illustrates this.

**Table 2:** Comparison Table between Arabic Datasets and Ours.

S/N	Name Database	MSA or dialect	Year	type	Size of database	Data format	Emotions
1	KSUEmotions	MSA (Yemen, Saudi Arabia, and Syria)	2017	acted	5 h and 10 min of recordings by 23 speakers	Audio	Neutral, happiness, sadness, surprise, anger
2	Egyptian Arabic speech emotion (EYASE) database	Egyptian dialect	2020	semi-natural	579 utterances by 3 male and 3 female professional actors from Egyptian TV series	Audio-visual	Anger, happiness, neutral, sadness
3	ANAD	Arabic	2018	Natural	1384 recordings from Arabic talk show	Audio	happiness, Anger, surprise
4	Arabic natural corpus	Arabic	2018	Natural	1384 recordings from online Arabic talk show	Audio	Anger, surprise, happiness
5	-	Algerian dialect	2019	Natural	1443 Sentences by14 speakers	audio-visual	Enthusiasm, admiration, disapproval, neutrality, and joy
6	-	Arabic	2020	Inductor	320 utterances by 32 speakers	-	Anger, fear, happiness and sadness
7	-	Saudi dialect	2021	Semi-natural	11 min 175 records from the popular Saudi YouTube channel Telfaz11	-	Anger, happiness, sadness, and neutrality
8	-	Arabic	2021	Not natural	200 Records by 10 speakers	Audio	Sadness, happiness, surprise, and questioning
9	EAED	Egyptian dialect	2023	semi-natural	From five different well-known Egyptian TV series.	-	happiness, sadness, anger, neutral, surprise, and fear
10	-	Arabic	2025	natural	2083 audio files	audio	anger, sadness, happiness, and neutral
11	YSED	Yemeni dialect	2025	Simulator and Inductor	1432 recordings by 68 speakers	Audio	Anger, happiness, neutral, sadness, fear

**Fig. 2:**
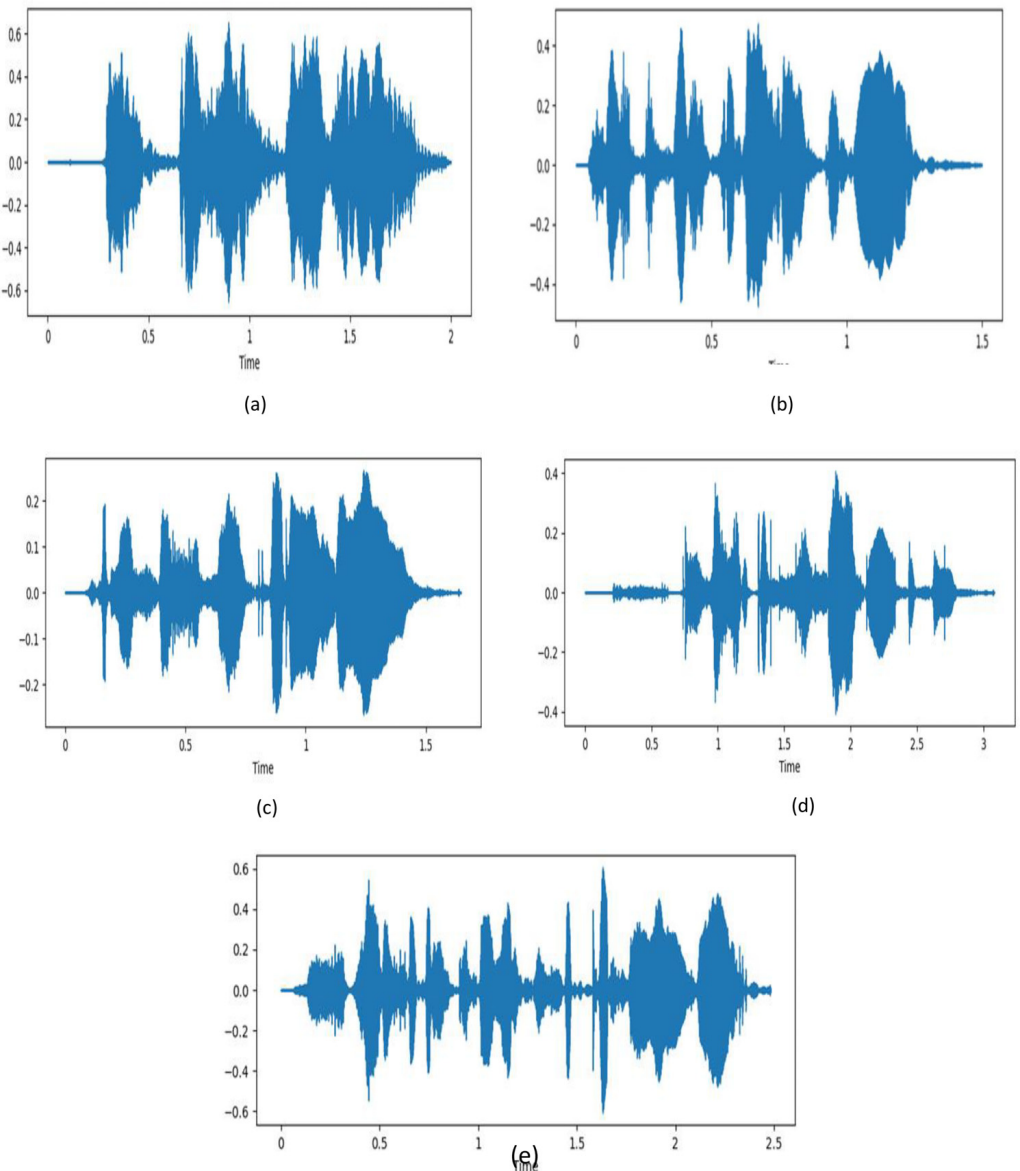
Waveform plot of a randomly selected sample of every emotional state, (a) angry, (b) happy, (c) neutral, (d) sad, and (e) fearful emotions of YSED dataset.

A waveform plot for every emotion of a randomly selected sample from the (YSER) dataset is presented in Fig. 2. Here, the X-axis is representing different time frames measured in seconds, and the Y-axis represents amplitude that indicates the amount of air compression (> zero) or rarefaction (< zero) induced by a moving object, such as the vocal cords, and pressure equilibrium point (= zero) denotes silence [16].

## 4 Experimental Design, Materials and Methods

**Fig. 3:**
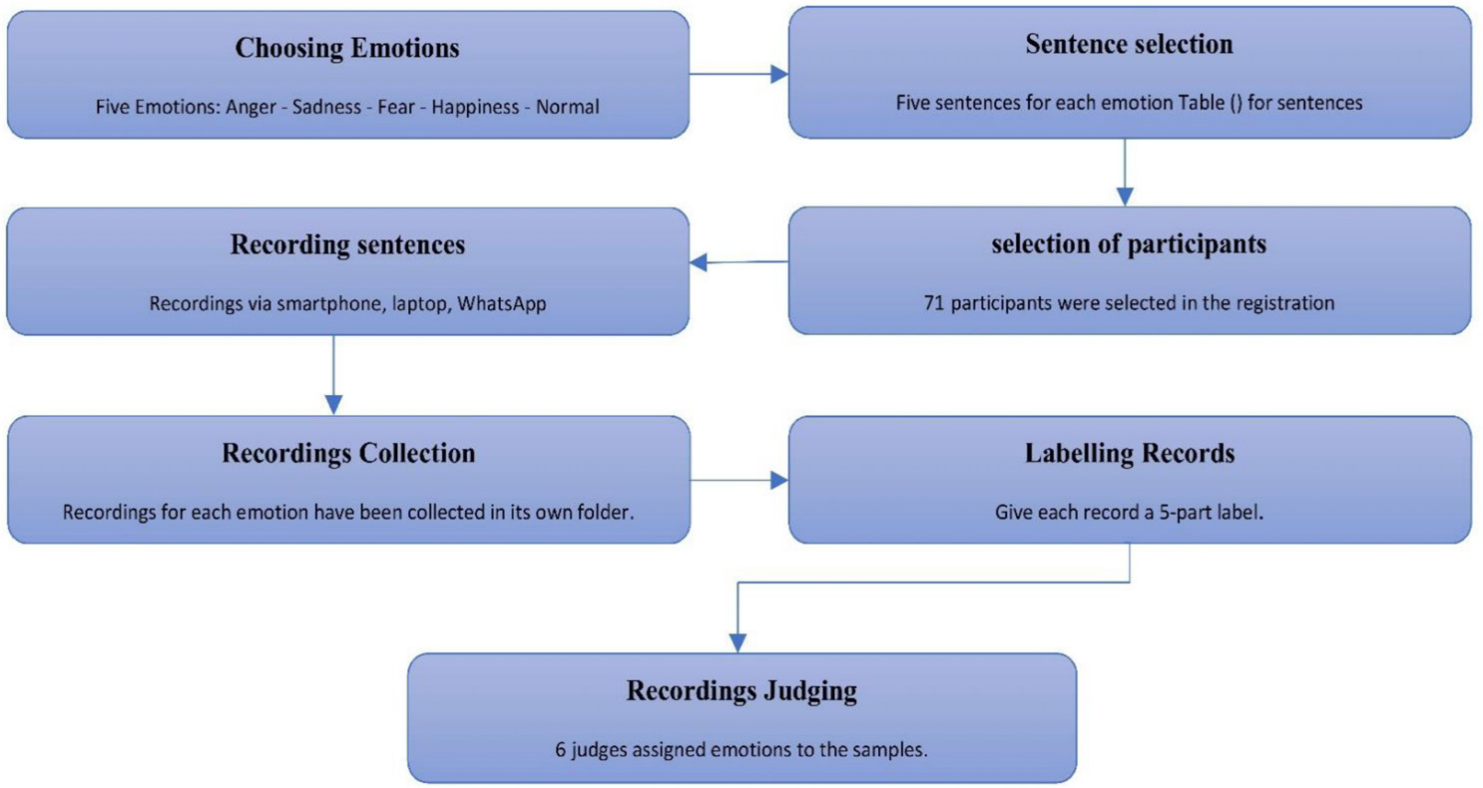
Workflow diagram of YSED dataset preparation.

The preparation of YSED dataset involves seven distinct steps: emotion selection, sentence selection, participant recruitment, sentence recording, record naming, recordings collection, recordings, and evaluation. This section provides a concise overview of each of these steps undertaken to develop YSED dataset. The workflow diagram illustrating YSED preparation process is presented in Fig. 3.

**1. Emotion Selection**

According to the theory of basic emotions [17], humans possess a specific set of basic emotions, such as anger, sadness, happiness, and fear.

We examined the Arabic dataset in Table 2 and discovered the emotions of neutral and surprise. Initially, we considered six basic emotions (happiness, fear, sadness, anger, surprise, and normal). However, because the tone of surprise is similar to both happiness and anger, these emotions may overlap. Therefore, we ignored surprise. Consequently, only the most prominent emotions were included, resulting in five emotions in the YSER dataset with an unbalanced number of records in each of these emotions.

**2. Sentence selection**

**Fig. 4:**
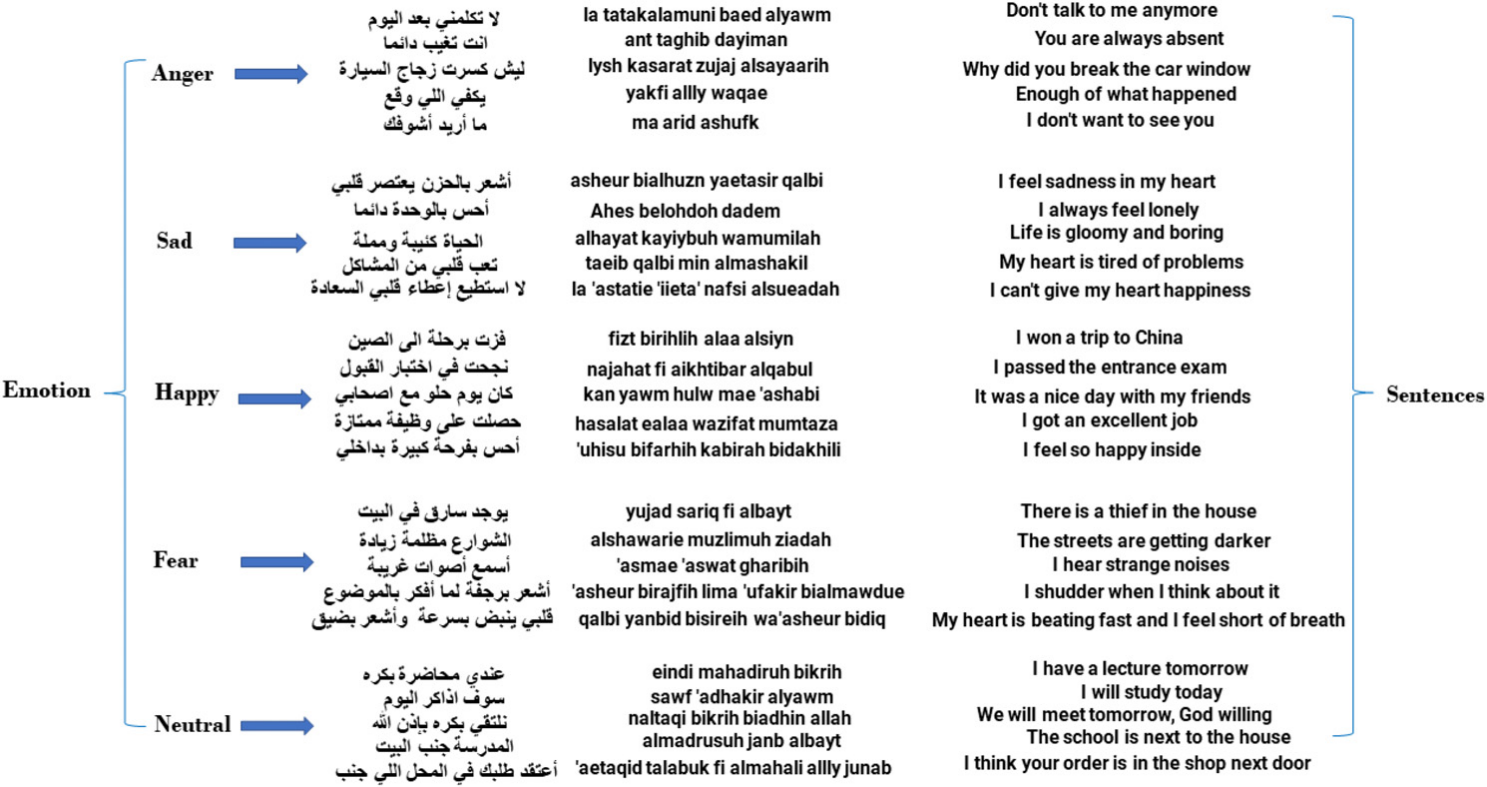
Shows the selected sentences for each emotion in the dataset (YSED).

Five sentences were selected for each emotion to ensure that they effectively convey the corresponding feelings. For instance, the sentence” I passed the admission test” was chosen to represent joy, while” I always feel lonely” exemplifies sadness. Additionally, neutral sentences, such as” I will study today,” were included to represent a none-motional state. In total, 25 sentences were curated (5 sentences for each of the five emotions). Fig. 4 presents the selected sentences categorized by emotion.

**Emotion generation approach**: Speech recordings for emotion recognition can be collected using three primary methods: simulated, induced, and natural. In the simulated approach, participants are instructed to read sentences while consciously expressing the target emotion. The induced approach involves prompting participants to experience the desired emotion before reading the sentence. Lastly, the natural approach entails recording participants when they naturally feel a specific emotion. In developing YSED dataset, the induced approach was employed for 80 % of the recordings, while the remaining 20 % utilized the simulated approach.

**3. Participant recruitment**

The dataset initially included 71 participants, all from the IBB Governorate and speaking the standard Yemeni dialect. These participants were divided into four categories: high school students, university students, teachers, and employees. To ensure they could effectively express a range of emotions, their abilities were assessed by a panel of judges.

During the evaluation phase, judges classified participants into three proficiency levels: professionals, semi-professionals, and amateurs, as detailed in Table 3. The participant who had clear experience in acting and could express emotion excellently was judged to be in the professional category. In contrast, the semi-professionals were judged to be those who could express emotion very well. Participants with extensive acting experience and exceptional emotional expression skills were designated as professionals. Those who could convey emotions very well but had limited experience were categorized as semi-professionals. Finally, participants without experience but deemed capable of adequately expressing emotions were assigned to the amateur category.

**Table 3:** Classification of the 68 participants who made YSED dataset recordings.

Category	Values
Occupation	High school students 19, University students 43, Government Teachers 6
Expertise	Professional (20), Semi-professional (30), Amateur (18)

Based on these assessments, the judges excluded three participants who were not deemed sufficiently capable to participate. Consequently, a total of 68 participants (37 males and 31 females) aged between 15 and 40 years were selected for inclusion in YSED dataset.

**4. Registration and data creation**

The preparation of phrases adhered to specific registration conditions and corresponding emotions, which were documented on paper. Additionally, a software application was developed using the C# programming language. This application integrated the selected sentences, the designated emotions, and registration conditions and provided an interface for recording audio.

These materials, including both the printed phrases and the C# application, were distributed to various schools, universities, and institutions. During these visits, the objectives and requirements of the registration process were clearly communicated to potential participants. The purpose of these interactions was to ensure that participants understood the study’s aims and the procedures involved. Following these presentations, the names of individuals interested in participating were collected and recorded for inclusion in YSED dataset.

A total of 71 participants contributed to YSED dataset. Recording sessions were conducted using various methods: some participants recorded the phrases at home with their mobile phones, others utilized specialized software applications, and a few recorded via WhatsApp. However, the majority of the recordings were captured using the researchers Galaxy A21 mobile phone equipped with a voice recorder application, due to the unavailability of professional recording equipment. Recordings were scheduled at times convenient for the participants. During each session, the researcher recorded the participants, who were then asked to re-record several times to ensure clarity and emotional authenticity. For each sentence, up to four recordings were obtained to capture the most representative emotional expression, except for neutral statements, which were recorded only once unless the initial recording was deemed incorrect.

Each participant was required to produce 85 recordings distributed across five emotions. Specifically, for the four primary emotions (anger, fear, happiness, and sadness), each participant recorded five sentences with four repetitions per sentence, resulting in 4 × 5 = 20 recordings per emotion. Each of the five sentences was recorded once for the neutral emotion, totalling five recordings. Consequently, the overall calculation for each participant is as follows: (20 recordings 4 emotions) + (5 recordings 1 emotion) = 85 recordings. With 71 participants involved, the dataset comprised 85 × 71=6035 audio recordings. Table 4 shows the emotions with the number of recording frequencies for each emotion.

**Table 4:** Emotions with repetition count.

Emotions	Number of Sentences	Number of Repetitions	Total Recordings
Happy	5	4	20
Fear	5	4	20
Angry	5	4	20
Sad	5	4	20
Neutral	5	1	5

Recordings were conducted at IBB University, College of Science, Department of Information Technology, in as quiet an environment as possible to minimize background noise. The distance between the speaker's mouth and the microphone was maintained at 25–30 cm to ensure optimal audio quality. After recording each sentence, the audio file was immediately saved. The researcher reviewed all submitted recordings to check their quality and emotional expression. If errors were detected, participants were asked to re-record the affected sentences to ensure the integrity and consistency of the dataset

**5. Files and tagging**

Emotions within the dataset were encoded using the following scheme: anger (a5), sadness (s4), happiness h3, fear f2, and neutral n1. Participant gender was designated as male 02 and female 01, with each participant assigned a unique identifier ranging from 1 to 68. Additionally, each recording included a repetition number (1–4) for each sentence, and the sentences themselves were numbered from 1 to 5.

Consequently, each audio file was named following the format n1–02–01–01–15.wav, which comprises five distinct components:

1. Emotion Code: Indicates the emotion expressed (e.g., n1 for neutral).
2. Sentence Number: Denotes the specific sentence recorded (e.g., 02).
3. Repetition Number: Represents the iteration of the recording for a particular sentence (e.g., 01).
4. Gender Code: Specifies the gender of the participant (e.g., 01 for female).
5. Participant Number: Identifies the unique participant (e.g., 15).

This structured naming convention ensures systematic organization and facilitates easy retrieval and analysis of the recordings within YSED dataset. The YSED dataset is freely available for research purposes.^1^

**6. Judges and Evaluation Process**

YSED dataset’s recordings were evaluated by a panel of six judges, all of whom are native Yemeni speakers fluent in the Yemeni dialect. The composition of the judging panel included diverse professional backgrounds: one faculty member from the University, one dentist, one accountant, one interior designer, one government school teacher, and one company employee.

To maintain objectivity, the judges were blinded to the target emotion associated with each recording. All six judges independently reviewed each audio recording and assessed whether the recording adequately expressed the intended emotion. In cases where the emotional expression was ambiguous, judges were instructed to reject the recording from being assigned to any of the five predefined emotions.

For recordings intended to convey a specific emotion, inclusion in YSED dataset required that at least four out of the six judges concur in accepting the recording as appropriately expressing that emotion. This consensus criterion ensured the reliability and validity of the emotional labels assigned to each recording.

Initially, the dataset was expected to comprise 6035 audio files, with 71 participants each contributing 85 recordings. However, following a rigorous selection process based on the judges' evaluations, many recordings were excluded due to insufficient agreement or unclear emotional expression. For instance, some judges classified the expression of anger as fear; the recording was excluded unless at least four judges agreed that the emotion was anger. In such cases, the recording was included in the anger category; otherwise, it was discarded. As a result, the final YSED dataset consists of 1432 validated recordings.

Inter-annotator agreement: To evaluate the consistency among the six judges, the Fleiss’ Kappa coefficient was utilized to measure the level of agreement beyond what would be expected by chance. The Fleiss’ Kappa is calculated using the following formula:

$$k=\frac{p_{o}-p_{e}}{1-p_{e}}$$

Where *p_o_* represents the observed agreement among the judges, and *p_e_* denotes the expected agreement by chance. *K* value of *1* indicates complete agreement among all raters [18].

For YSED dataset, the Fleiss’ Kappa coefficient was calculated to be *0.9*. This high Kappa value signifies substantial agreement among the six judges, demonstrating the reliability and consistency of the emotional annotations within the dataset. Such a high level of inter-annotator agreement underscores the robustness of the evaluation process and the quality of YSED dataset, making it a dependable resource for subsequent emotion recognition research in Yemeni Arabic*.*

## Limitations

As a team, we faced problems, including the inability to purchase high-quality recording tools and equipment due to their high prices, and the inability to record in private studios. Moreover, we found it difficult to gather a large number of participants, as most of them refrained from participating due to the privacy of Yemeni society and the lack of previous experiences of the same type, as far as we know. Also, the judging team took time to judge all the recordings due to the number of recordings and the need to classify them.

## Ethics Statement

This research was conducted in accordance with the highest ethical standards of scientific research, while ensuring the rights and safety of participants. The research team obtained written informed consent from all adult participants, while consent was obtained from the legal guardians of minor participants. The IBB University Research Ethics Committee verified these consents, and all procedures were in compliance with approved ethical principles.

Ethical Procedure Details:

- Informed Consent: The researchers provided a clear explanation of the research objectives and the importance of participation, emphasizing that participation was voluntary and without any coercion.
- Right to Withdraw: Participants retained the freedom to withdraw their consent or discontinue their participation at any stage of the research.
- Privacy Protection: All recordings were collected anonymously to ensure anonymity, and data were treated with complete confidentiality after collection.
- Final Consent: Participants gave their explicit consent to the use of their data after recording was completed.

The research team affirms its full commitment to ensuring the integrity of the research and respecting the rights of participants in accordance with international standards of scientific ethics.

## Credit Author Statement

**Somia Derhem:** Conceptualization, methodology, data Construction, writing, original Draft. **Eiad AL-Mekhlafi:** Conceptualization, supervision, Review and Editing. **Nashwan Ahmed AL-Majmar:** Conceptualization, and supervision. **Moeen AL-Makhlafi:** Writing, Review and Editing.

## Data Availability

zenodoYSED: First edition of Yemeni speech's emotions (Original data)

## Footnotes

1 https://zenodo.org/records/15227219.
